# The Role of Inflammation in Takotsubo Syndrome: From Pathogenic Pathways To Imaging Insights and Therapeutic Perspectives

**DOI:** 10.1007/s11886-025-02342-4

**Published:** 2026-02-04

**Authors:** Cristina Madaudo, Hibba Kurdi, Jessica Ielapi, Cornelia Margineanu, Sara Moscatelli, Chiara Bucciarelli-Ducci, Daniel Bromage, Jessica Artico

**Affiliations:** 1 Department of Health Promotion, Mother and Child Care, Internal Medicine and Medical Specialties (ProMISE), University of Palermo, Palermo, Italy; 2 Institute of Cardiovascular Science, University College London, Gower Street, WC1E 6BT London, United Kingdom; 3 Department of Experimental and Clinical Medicine, Magna Graecia University, Viale Europa, Catanzaro, 88100 Italy; 4 University of Medicine and Pharmacy Carol Davila, Bucharest, Romania; 5 Inherited Cardiovascular Diseases, Great Ormond Street Hospital, Children’s NHS Foundation Trust, London, UK; 6 Royal Brompton and Harefield Hospitals, Guys & St Thomas NHS Trust, London, United Kingdom; School of Biomedical Engineering and Imaging Sciences, Faculty of Life Sciences and Medicine, Kings College University, London, UK; 7 British Heart Foundation Centre of Research Excellence, School of Cardiovascular Medicine, Faculty of Life Science, King’s College London, London, UK; 8 Imperial College Healthcare NHS Trust, London, United Kingdom; National Heart and Lung Institute, Imperial College London, Hammersmith Hospital, Du Cane Road, London, W12 0HS UK

**Keywords:** Takotsubo syndrome, Stress-induced cardiomyopathy, Inflammation, Pathophysiology, Imaging, Therapeutic targets

## Abstract

**Purpose of Review:**

Takotsubo syndrome (TTS), also known as stress-induced cardiomyopathy, is an acute and transient form of myocardial dysfunction that predominantly affects postmenopausal women after emotional or physical stress. Although initially considered benign, growing evidence demonstrates that TTS carries substantial morbidity, recurrence, and mortality risks. This review aims to summarize current knowledge on the pathophysiology of TTS with a focus on inflammation, to explore the interplay between stress and myocardial injury, and to discuss the diagnostic and prognostic value of multimodality imaging together with emerging therapeutic approaches, providing a comprehensive framework for clinical practice and future research.

**Recent Findings:**

The pathophysiology of TTS is multifactorial, involving sympathetic hyperactivation with catecholamine excess, microvascular dysfunction, epicardial coronary spasm, intracellular calcium overload, and myocardial stunning. Increasing evidence supports a central role of inflammation, including activation of the NLRP3 inflammasome, release of cytokines such as IL-1β, IL-6, and TNF-α, oxidative stress, and macrophage polarization, ultimately leading to myocardial injury, fibrosis, and adverse ventricular remodelling. Multimodality imaging, comprising echocardiography, cardiac magnetic resonance, and nuclear techniques, enables early identification of functional and structural abnormalities, exclusion of differential diagnoses such as acute coronary syndromes or myocarditis, and prognostic assessment.

**Summary:**

TTS represents a complex stress-related cardiomyopathy with overlapping neurohormonal, inflammatory, and microvascular mechanisms. Current management remains largely supportive, focused on heart failure therapy, anticoagulation when indicated, and hemodynamic stabilization. Novel therapies targeting inflammatory and sympathetic pathways are under investigation and may change the future management of this condition. Understanding the interplay between stress, inflammation, and myocardial injury offers new opportunities for pathophysiology-driven treatment strategies and improved patient outcomes.

**Graphical Abstract:**

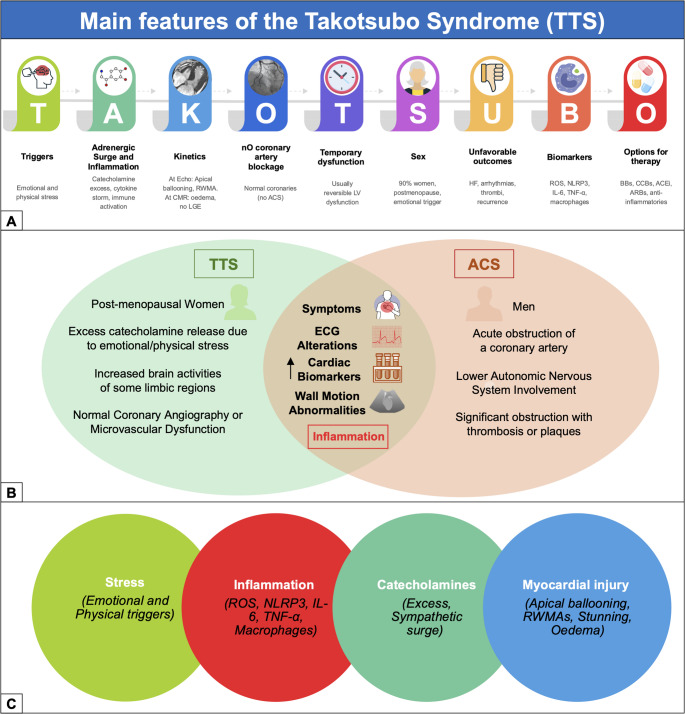

## Introduction

 Takotsubo syndrome (TTS) is an acute, typically transient myocardial dysfunction, often preceded by significant emotional or physical stress triggers [1–6]. First described in 1990 by Sato et al., the condition derives its name from the similarity between Japanese octopus’ pots, with a round base and narrow neck, and the characteristic shape of the left ventricle (LV) in affected individuals [1, 7].

Clinically, TTS often mimics acute coronary syndromes (ACS), with similar symptoms (such as chest pain and dyspnoea), ECG changes, and regional wall motion abnormalities (RWMAs) [1, 8–10] (Central Illustration). However, TTS typically presents in the absence of significant coronary artery disease (CAD) [1, 8, 10, 11]. TTS accounts for approximately 1–3% of patients initially suspected of having ACS, with an incidence ranging from 15 to 30 cases per 100,000 person-years and a mean age at presentation between 62 and 76 years [8, 12–15]. Occurring predominantly in women, especially in the postmenopausal period, its prevalence rises to 6–9% in this subgroup, which represents around 90% of all cases [2, 16, 17]. Although men are less frequently affected, they tend to experience more severe complications and higher mortality [2, 18, 19]. The most common variant, characterized by apical ballooning, accounts for approximately 80% of all cases [2, 20], followed by mid-ventricular (15%) and basal/inverted forms (2%) [21–24]. Rarely, focal, biventricular, or isolated right ventricle (RV) dysfunction may occur [21, 25, 26]. TTS, though often considered benign, may rarely lead to acute complications like heart failure (HF), arrhythmias, LV outflow obstruction, thromboembolism, as well as left ventricular free wall rupture, with an overall in-hospital mortality between 0 and 8% [9, 10, 14, 27]. Although multiple studies have proposed that TTS is primarily triggered by a catecholamine surge in response to acute emotional or physical stress, through activation of the hypothalamic–pituitary–adrenal (HPA) axis, this hypothesis accounts for only part of the pathophysiological complexity of the syndrome [28]. Catecholamines, particularly epinephrine and norepinephrine, may exert direct toxic effects on the myocardium, including transient myocardial stunning, impaired perfusion, cellular damage, and metabolic dysregulation [29, 30]. However, more recent evidence demonstrates that circulating epinephrine levels are usually normal or only mildly elevated, and similar elevations occur in several acute medical conditions such as sepsis, anaphylaxis and hypoxemia without inducing TTS [31]. This suggests that epinephrine excess alone is insufficient to explain TTS, which likely results from the interaction of sympathetic activation with additional mechanisms such as microvascular dysfunction, autonomic imbalance and inflammatory signaling [31].

The LV apex is particularly vulnerable, likely due to a higher β2-to-β1 adrenergic receptor ratio, which increases sensitivity to catecholamine-induced injury [32]. Other contributory mechanisms include diffuse coronary vasospasm, microvascular dysfunction, and altered endothelial reactivity, especially in postmenopausal women with reduced oestrogen levels [33–35]. Accumulating evidence suggests that these additional mechanisms may either potentiate catecholamine-mediated injury or, in some patients, represent independent pathogenic pathways capable of reproducing the characteristic pattern of transient ventricular dysfunction [36]. Genetic predisposition and anatomical variations in the sympathetic nervous system may also play a role, as suggested by familial clustering of TTS cases [37–40]. Experimental evidence supporting stress-related neuro-cardiac mechanisms remains limited, as animal models that accurately reproduce TTS pathophysiology are scarce. One of the few models incorporating the emotional-stress component is the rat immobilization model, in which acute fear-related stress triggered transient LV hypocontractility, including apical ballooning, via activation of cardiac adrenoceptors in the absence of ischemia-reperfusion injury [41]. Pretreatment with α- and β-adrenoceptor blockade normalized wall-motion abnormalities, supporting a neuro–cardiac stress mechanism rather than direct ischemic damage [41]. Evidence in humans is even more restricted. Although alterations in central autonomic networks have been proposed, current data supporting a brain–heart axis are limited, mostly arising from small neuroimaging studies or indirect autonomic markers [42–44]. Direct demonstration of central neural involvement in TTS is still lacking and will require dedicated neuro-cardiac imaging, autonomic testing, and longitudinal studies specifically designed to assess brain–heart interactions [44].

Current evidence on inflammation suggest its central role in the pathophysiology of TTS [45]. Given the emerging evidence linking inflammation to TTS onset and progression, this review aims to clarify and explore the role of inflammatory mechanisms in its development and clinical course.

## Inflammation in TTS

**Fig. 1:**
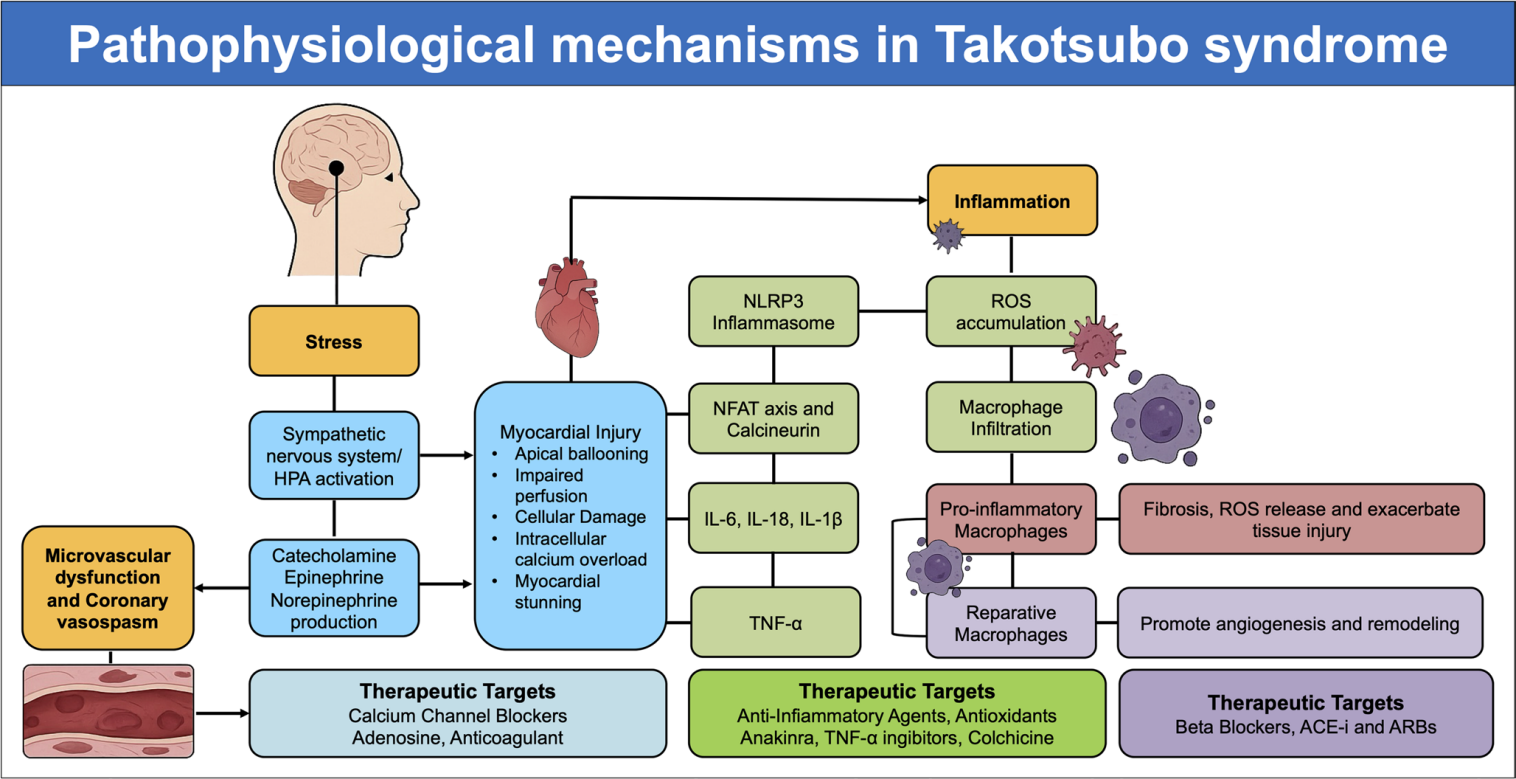
Pathophysiological mechanisms linking stress to myocardial injury and remodeling in TTS. Psychological or physical stress triggers activation of the sympathetic nervous system and the HPA axis, leading to catecholamine release (epinephrine, norepinephrine). Excess catecholamines contribute to microvascular dysfunction and epicardial coronary vasospasm, as well as direct cardiotoxic effects mediated by intracellular calcium overload and activation of the calcineurin–NFAT signaling pathway, ultimately resulting in myocardial stunning and injury (apical ballooning, impaired perfusion, and cellular damage). Myocardial injury activates inflammatory pathways, with NLRP3 inflammasome activation leading to IL-1β and IL-18 release, followed by increased IL-6 and TNF-α expression, generation of reactive oxygen species (ROS), and macrophage infiltration with M1→M2 polarization. These mechanisms promote fibrosis, angiogenesis, and adverse ventricular remodeling. Potential therapeutic targets are highlighted at each step of the cascade, including calcium channel blockers, adenosine, anticoagulants, anti-inflammatory agents (e.g., anakinra, TNF-α inhibitors, colchicine), antioxidants, β-blockers, ACE inhibitors, and ARBs. Abbreviations: ACEi, angiotensin-converting enzyme inhibitors; ARBs, angiotensin receptor blockers; HPA, hypothalamic–pituitary–adrenal; IL, interleukin; NFAT, nuclear factor of activated T cells; NLRP3, NOD-, LRR- and pyrin domain-containing protein 3; ROS, reactive oxygen species; TNF, tumor necrosis factor; TTS: Takotsubo syndrome

Immune activation and inflammation have emerged as critical contributors to the pathophysiology of TTS [45–47]. Whether inflammation represents a primary driver of TTS or a secondary response to acute neurohormonal and microvascular stress remains uncertain. The interplay between sympathetic overstimulation, microvascular dysfunction, and inflammatory pathways is summarized in Fig. 1, which outlines the cascade from stress-induced catecholamine release to myocardial injury, inflammasome activation, cytokine release, and subsequent fibrosis and ventricular remodelling.

Excessive sympathetic stimulation activates the NOD-, LRR- and pyrin domain-containing protein 3 (NLRP3) inflammasome in cardiomyocytes, prompting the release of interleukins (IL), such as IL-1 β, IL-6 and IL-18, and promoting galectin-3-mediated macrophage infiltration [46, 47]. Additionally, NOX4-derived mitochondrial reactive oxygen species (ROS) amplify the reaction, leading to cardiac inflammation, microscopic fibrosis, and myocardial dysfunction [46, 48]. The interplay between acute neurohormonal stress, oxidative damage, and activation of innate immune pathways triggers a rapid and sustained inflammatory response, characterized by cytokine release, chemokine-induced leukocyte recruitment, and macrophage-dominated myocardial infiltration [48]. Specific intracellular signalling cascades, such as the calcineurin-Nuclear Factor of Activated T-cells (NFAT) axis, further amplify this process. At the same time, the balance between pro-inflammatory and reparative macrophage phenotypes appears to influence long-term myocardial recovery and ventricular remodelling [49, 50]. Understanding inflammatory activation in TTS is crucial not only for elucidating its pathogenesis but also for improving patient risk stratification and guiding tailored therapies. Although elevated cytokines, macrophage infiltration, and oxidative stress are consistently observed in TTS, similar inflammatory signatures occur in acute myocardial infarction, myocarditis and sepsis, yet these conditions do not reproduce the characteristic pattern of transient regional dysfunction seen in TTS. As seen in other cardiac diseases, such as myocarditis, recognising and characterising inflammation may provide a key to identifying patients at higher risk of adverse outcomes and to developing targeted treatment approaches [51, 52]. A recent study further supported this concept, showing that systemic inflammatory markers such as the neutrophil-to-lymphocyte ratio (NLR) and lymphocyte-to-monocyte ratio (LMR) are independently associated with prognosis in TTS [53]. In that analysis, patients with TTS displayed higher monocyte counts and lower LMR compared with those with acute coronary syndromes, and both NLR and LMR predicted long-term mortality, underscoring the contribution of systemic inflammation to adverse outcomes [53].

A brief overview of the main mechanisms, their molecular mediators, supporting evidence, and potential therapeutic targets is provided in Table 1. Inflammation alone is likely insufficient to explain the syndrome and may instead act as an amplifier of myocardial injury. Therefore, clarifying the causal role of inflammation will require dedicated prospective studies and interventional randomized clinical trials (RCTs) specifically designed to test whether modulating inflammatory pathways can influence the course of TTS.

**Table 1:** Summary of inflammatory mechanisms in Takotsubo syndrome

	Key Mechanisms	Mediators	Clinical/Experimental Evidence	Potential Therapeutic Targets
Stress–Inflammation Relationship	Stress-induced activation of HPA axis and sympathetic nervous system; catecholamine surge; oxidative stress via NADPH oxidase (NOX4); activation of NLRP3 inflammasome and calcineurin–NFAT signalling	Catecholamines, ROS, NOX4, NLRP3, calcineurin	Animal models and patient data show that catecholamine excess triggers oxidative stress and inflammatory pathways	Antioxidants, NADPH oxidase inhibitors, inflammasome inhibitors, and calcineurin inhibitors
Acute Inflammatory Response	Cytokine storm with pro- and anti-inflammatory components; chemokine-driven leukocyte recruitment	IL-6, IL-18, TNF-α, IL-1β, IL-10, MCP-1	Elevated cytokines in acute TTS; correlation between IL-6 levels and LV dysfunction severity	Anti-cytokine therapy (IL-1 or IL-6 blockade), chemokine antagonists
Myocardial Infiltration	Influx of mononuclear cells, mainly macrophages, into myocardial interstitium; interaction with cardiomyocytes and fibroblasts	Macrophages and monocytes	Histopathology showing macrophage-rich infiltrates; CMR evidence of oedema; animal models confirming early infiltration	Modulators of macrophage polarization, immunomodulatory agents
Calcineurin Signalling	Calcium/calmodulin-dependent phosphatase activation; NFAT dephosphorylation and nuclear translocation; transcription of inflammatory genes	Calcineurin, NFAT	Experimental data linking calcineurin activation to sustained inflammation; inhibition reduces injury in models	Calcineurin inhibitors (e.g., cyclosporine), NFAT pathway modulators
Pro-inflammatory Cytokines & Macrophages	Pro inflammatory/reparative macrophages balance regulates injury vs. repair; cytokines sustain inflammation or promote resolution	IL-6, IL-18, TNF-α, IL-1β; pro-inflammatory/ reparative macrophages	High IL-6 linked to worse outcomes; Pro inflammatory macrophages dominance prolongs injury, reparative macrophages support healing	Therapies promoting reparative macrophages polarization, cytokine blockade, and resolution-phase mediators

Key: CMR: cardiac magnetic resonance; HPA: hypothalamic–pituitary–adrenal; IL: interleukin; LV: left ventricle; MCP-1: monocyte chemoattractant protein 1; NFAT: nuclear factor of activated T cells; NLRP3: NOD-, LRR- and pyrin domain-containing protein 3; NOX4: NADPH oxidase isoform 4; ROS: reactive oxygen species; TNF-α: tumor necrosis factor alpha; TTS: Takotsubo syndrome

### Stress–Inflammation Relationship

Acute emotional or physical stress, the hallmark trigger of TTS, initiates a complex cascade that extends well beyond transient catecholamine-mediated myocardial stunning to involve an activation of inflammatory pathways [28, 54]. Stress-induced stimulation of the HPA axis and sympathetic nervous system results in an abrupt and massive catecholamine surge. This neurohormonal storm has direct cardiotoxic effects via β-adrenergic receptor overstimulation, intracellular calcium overload, and mitochondrial dysfunction [55]. In parallel, excessive catecholamine exposure promotes oxidative stress through activation of Nicotinamide Adenine Dinucleotide Phosphate (NADP) oxidases, particularly NOX4, leading to the accumulation of ROS [56]. These ROS act as potent activators of redox-sensitive intracellular signalling pathways, including the NLRP3 inflammasome and the calcineurin–nuclear factor of activated T-cells (NFAT) axis [57]. The result is an amplification loop in which oxidative stress and inflammation sustain each other, potentially prolonging myocardial dysfunction beyond the period of hemodynamic recovery. Experimental evidence suggests that blocking ROS generation or downstream inflammatory signalling may attenuate myocardial injury in stress-induced cardiomyopathy models [58]. Importantly, the inflammatory response does not occur in isolation but interacts closely with other proposed pathogenic mechanisms of Takotsubo syndrome. Elevated catecholamines and oxidative stress impair endothelial nitric oxide bioavailability, promoting coronary microvascular dysfunction and diffuse vasospasm [58]. Endothelial activation further enhances leukocyte adhesion and cytokine release, reinforcing local inflammation and vascular dysregulation. In postmenopausal women, reduced oestrogen levels exacerbate these effects by diminishing endothelial resilience and modulating β-adrenergic receptor sensitivity [59]. Thus, the stress–inflammation axis is tightly interwoven with microvascular and endothelial pathways, suggesting that TTS may arise from a convergence of neurohormonal, vascular, and inflammatory perturbations rather than a single dominant mechanism.

### Acute Inflammatory Response

During the acute phase of TTS, patients display a distinct systemic inflammatory profile. Circulating levels of pro-inflammatory cytokines, including IL-6, IL-18, tumor necrosis factor-α (TNF-α), and in some cases IL-1β are significantly elevated compared with healthy controls and patients with ACS [60, 61]. This cytokine surge often peaks within the first 48–72 h and then gradually declines, mirroring the acute clinical course. Interestingly, anti-inflammatory cytokines -such as IL-10- also increase, possibly as a compensatory attempt to limit immune-mediated damage [62]. Chemokines, including monocyte chemoattractant protein-1 (MCP-1), are upregulated early, promoting the recruitment of circulating monocytes to the myocardium [63]. The combination of high pro-inflammatory cytokines and chemokine-driven leukocyte recruitment creates a myocardial microenvironment primed for immune cell infiltration and local inflammatory damage [64]. Serial measurements suggest that the magnitude of IL-6 elevation may correlate with the severity of LV dysfunction and could serve as a biomarker for prognosis [65]. 

### Myocardial Infiltration

Histopathological findings from post-mortem studies and endomyocardial biopsies have provided direct evidence of myocardial inflammation in TTS [66]. The myocardium in the acute and subacute phases often shows infiltration by mononuclear inflammatory cells, with monocytes/macrophages being the predominant type [50]. These cells are distributed both within the interstitium and close to damaged cardiomyocytes, where they can interact with fibroblasts and endothelial cells to promote fibrosis [50]. Imaging studies, particularly cardiac magnetic resonance (CMR), corroborate these pathological findings by revealing myocardial oedema [67]. Experimental data indicate that myocardial infiltration begins within hours of stress exposure and can persist for several days, contributing to the persistence of RWMAs even after partial recovery of contractile function [68]. Although these inflammatory features are not unique to TTS and are observed in various forms of myocardial injury, several aspects appear distinctive. The inflammatory infiltrate in TTS is typically modest in intensity, lacks the neutrophilic component characteristic of acute ischemic necrosis, and occurs in the absence of significant myocyte death [45]. Moreover, the regional distribution of inflammation parallels the pattern of wall-motion abnormalities rather than coronary territories, suggesting a neurohumorally mediated, reversible injury rather than infarction. Macrophage phenotyping studies have also shown a predominance of pro-inflammatory macrophages acutely, followed by a delayed shift toward reparative phenotypes, which may underlie the typically favorable recovery pattern [48]. Collectively, these observations suggest that myocardial infiltration in TTS represents a distinct, self-limited inflammatory reaction to catecholamine toxicity and microvascular dysfunction, rather than a classical myocarditic or ischemic process [48]. 

### Calcineurin Signalling

Calcineurin, a calcium/calmodulin-dependent phosphatase, emerges as a key molecular link between catecholamine excess, calcium dysregulation, and inflammatory gene activation [69]. Under oxidative and mechanical stress, calcineurin becomes activated, leading to dephosphorylation of Nuclear Factor of Activated T-cells (NFAT) transcription factors, which then translocate to the nucleus [70, 71]. NFAT promotes transcription of genes encoding pro-inflammatory cytokines, chemokines, and adhesion molecules [71]. In preclinical models, calcineurin inhibition has been shown to dampen inflammatory gene expression, reduce immune cell infiltration, and mitigate structural damage [72, 73]. This suggests that calcineurin may represent a promising therapeutic target for modulating the inflammatory component of TTS [74]. Recent evidence has expanded the inflammatory paradigm of TTS beyond the myocardium. Ziegler et al. recently described, for the first time, ganglionic inflammation involving cardiac sympathetic ganglia in a patient with TTS, characterized by macrophage infiltration and pro-inflammatory gene activation confirmed by single-nucleus RNA sequencing and experimental models. These findings suggest that neuroinflammation within the sympathetic chain may contribute to autonomic dysregulation and catecholamine hypersensitivity in TTS, providing a novel mechanistic link between stress, inflammation, and myocardial injury [75]. 

### Pro-inflammatory Cytokines and Role of Macrophages

The pro-inflammatory cytokine profile in TTS plays a dual role: initiating tissue injury and orchestrating repair. IL-6, IL-18, TNF-α, and IL-1β contribute to endothelial activation, increased vascular permeability, and further recruitment of leukocytes [48, 49]. Elevated IL-6, in particular, has been linked to worse in-hospital outcomes and delayed recovery [76]. Macrophages dominate the myocardial inflammatory infiltrate, displaying a spectrum of activation states. Pro-inflammatory macrophages, stimulated by interferon-γ and danger-associated molecular patterns (DAMPs), release ROS, nitric oxide, and pro-inflammatory cytokines that exacerbate tissue injury [49]. Reparative macrophages, induced by IL-4 and IL-13, facilitate resolution of inflammation, promote angiogenesis, and support extracellular matrix remodelling [49, 77]. The temporal shift from pro-inflammatory to reparative dominance appears to be a determinant of recovery trajectory, with a prolonged pro-inflammatory phenotype potentially contributing to residual fibrosis and a higher risk of recurrence [49, 78]. What seems distinctive in TTS, however, is the transient, spatially confined, and largely sterile nature of this inflammatory response. In contrast to ischemic or myocarditic injury, macrophage infiltration occurs without significant myocyte necrosis and mirrors the regional pattern of wall-motion abnormalities rather than coronary perfusion territories [47]. Moreover, the rapid phenotypic switching from pro-inflammatory to reparative macrophages as catecholamine levels normalize may underlie the characteristic reversibility of myocardial dysfunction [79]. Understanding the molecular cues that govern this tightly regulated inflammatory cycle, potentially involving catecholamine signaling, endothelial-derived mediators, and specialized pro-resolving lipid molecules, could open avenues for targeted therapies aimed at accelerating inflammation resolution without impairing necessary repair processes.

## Imaging

**Fig. 2:**
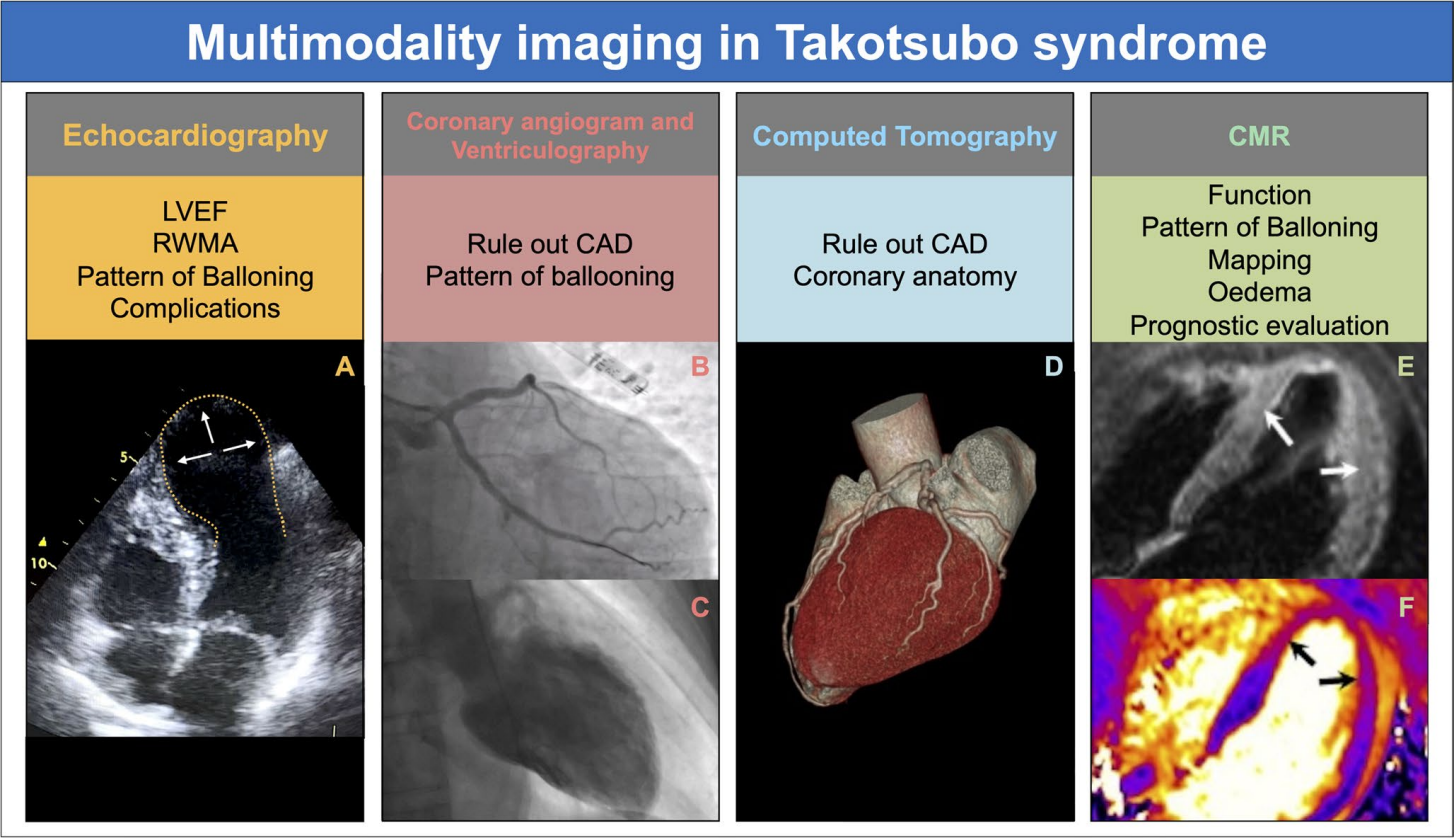
Multimodality imaging in Takotsubo syndrome (TTS). **Panel A**: Transthoracic echocardiography (apical 4-chamber view) showing the classic apical ballooning pattern with basal hyperkinesia, reduced LVEF, and regional wall motion abnormalities (RWMAs). The *white arrows* indicate the akinetic apical segments, while the *yellow dotted line* outlines the ballooning contour of the left ventricle **Panels B and C**: Coronary angiography excluding obstructive coronary artery disease (Panel B), followed by left ventriculography (Panel C) demonstrating the typical apical akinesis with basal hypercontractility **Panel D**: Cardiac CT illustrating coronary anatomy without significant stenosis and allowing functional assessment **Panel E**: CMR long-axis 4-chamber T2-STIR sequence showing areas of increased signal intensity consistent with myocardial oedema (*white arrows*), without late gadolinium enhancement (LGE) **Panel F**: CMR long-axis 4-chamber T2 mapping demonstrating elevated T2 values, consistent with myocardial oedema. The *black arrows* indicate regions of increased T2 signal intensity within the apical and mid-ventricular myocardium, corresponding to areas of myocardial oedema, useful for tissue characterization and prognostic evaluation (**Panels E and F** of Fig. 2 were reproduced with permission from: Arcari L. et al. Cardiac magnetic resonance in patients with Takotsubo syndrome: Clinical correlates of T2 mapping. Int J Cardiol. 2025 Jan 15;419:132716. © Elsevier [2025]) [86]. **Abbreviations:**CAD, coronary artery disease; CMR, cardiac magnetic resonance; CT, computed tomography; LGE, late gadolinium enhancement; LVEF, left ventricular ejection fraction; RWMAs, regional wall motion abnormalities; STIR, short tau inversion recovery; TTS, Takotsubo syndrome

Multimodality imaging plays a central role in the diagnosis, prognostic prediction and follow-up of patients with TTS **(**Fig. 2**).** [80, 81] Because the initial presentation often mimics ACS, the immediate priority is to rule out obstructive CAD according to pre-test probability typically with invasive coronary angiography in higher-risk presentations or ongoing ischaemia, and coronary computed tomography (CT) angiography (CTCA) as a non-invasive alternative in appropriately selected stable patients. Once obstructive CAD has been reasonably excluded, a multimodality pathway with TTE as first-line, CMR for tissue characterisation, and CT/nuclear techniques in selected cases adds substantial incremental value for diagnostic confirmation, risk stratification, and longitudinal follow-up [80, 81].

### Echocardiography

Transthoracic echocardiography (TTE) is usually the first-line imaging modality in suspected TTS, often from the initial emergency assessment [82]. The baseline study should document overall LV systolic function, RWMAs, possible RV involvement, the presence and haemodynamic impact of LV outflow tract (LVOT) obstruction and its effect on the mitral valve, along with a characterization of the degree of mitral regurgitation (MR), when present [82]. Typical echocardiographic features include apical ballooning with hypercontractile basal segments and RWMAs not corresponding to a single coronary artery territory. When image quality allows, 3-dimensional (3D) quantification of LVEF is preferred over the biplane method, and LV strain should be measured because reduced global longitudinal strain (GLS) carries prognostic information in TTS [83]. 

In the apical variant, careful screening for LV apical thrombus is recommended. Echocardiography is also the modality of choice for routine follow-up, where most patients show progressive normalisation of LV function [8, 80, 82, 84]. Given the small but serious risk of mechanical complications, the pericardium and colour/contrast Doppler flows should be scrutinised to exclude left-ventricular free-wall rupture [27]. 

### Ventriculography

Although other imaging techniques are more sensitive for detecting myocardial oedema and inflammation, many centres perform ventriculography in the context of patients with a STEMI-like clinical presentation, during a invasive coronary angiography, which in most cases rules out acute CAD [85]. The presence of coronary atherosclerosis is not an uncommon finding, but does not account for the observed RWMAs. When performed, ventriculography can identify characteristic TTS patterns, assess systolic function, detect mechanical complications, and allow invasive measurement of the pressure gradient by withdrawing the catheter from the LV cavity into the aorta. Complete opacification of the LV is typically sufficient to detect RWMAs and their distribution [80]. 

However, given the availability and superior tissue characterization of CMR, ventriculography is generally reserved for patients who undergo angiography as part of the initial exclusion of ACS.

### Cardiac Magnetic Resonance

CMR is considered the gold standard for assessing cardiac function and for tissue characterization [86]. In fact, its ability to interact with hydrogen protons through various imaging sequences makes it the modality of choice for detecting myocardial inflammation and oedema [87]. 

Balanced steady-state free precession (b-SSFP) sequences are acquired in long-axis views (2-, 3-, and 4-chamber) and in multiple short-axis slices covering the entire heart. RWMAs, visible in orthogonal planes, help identify the characteristic patterns of TTS and may reveal associated features such as LVOT flow acceleration or MR. Biventricular LVEF is calculated using the stack-of-discs method. Myocardial thickening due to oedema can also often be appreciated on cine images.

The main advantage of CMR is its ability to provide detailed structural characterization through the combined interpretation of multiple imaging sequences [88]. Importantly, significant diagnostic information can be obtained without contrast administration [89]. 

On T2-weighted sequences, oedematous regions appear hyperintense [90]. T1 and T2 mapping values in affected areas are moderately to markedly elevated, allowing for clear differentiation from normal myocardium, which shows normal values [91]. Visual mapping of these parameters is highly suggestive of TTS. In the acute phase, a typical finding is an apical-to-basal gradient in T1/T2 mapping (and oedema burden) that mirrors the regional dysfunction [92]. 

Consistently, Aikawa et al. confirmed a basal-to-apical increase in myocardial T2 relaxation times, with shortening toward normal values at ~ 3-month follow-up [93]. 

Following contrast administration, early gadolinium enhancement (EGE) sequences—acquired 1 to 3 min post-injection—can detect intraventricular thrombi, that could be located at the LV apex in apical variants. A hallmark of TTS is the absence of LGE on delayed imaging. However, in few cases with extensive myocardial oedema and inflammation, LGE may be present. This atypical finding is associated with lower rates of LV systolic function recovery and generally worse clinical outcomes [94]. On follow-up CMR, most patients show normalisation of LV systolic function with regression of oedema and return of T1/T2 mapping values towards the normal range [88]. This typically occurs within 3 months, although partial improvement can already be observed after 4–6 weeks [88]. In some cases, subtle abnormalities in myocardial tissue characteristics or strain parameters may persist beyond 3–6 months, suggesting incomplete resolution of subclinical inflammation or residual myocardial remodelling [88]. 

### Computer Tomography

The role of CT in TTS primarily involves the exclusion of other causes of acute chest pain, such as CAD, pulmonary embolism, or aortic pathology. Given the frequent ACS-like presentation, clinicians should also consider ruling out CAD according to pre-test probability: in stable, appropriately selected patients, CTCA can non-invasively exclude obstructive disease, whereas invasive coronary angiography remains preferred in higher-risk presentations or ongoing ischaemia [80, 95]. 

However, beyond its conventional diagnostic use, there are reported cases where CT has provided additional insights into RWMAs, using systolic and diastolic phase acquisitions, albeit with the limitation of increased radiation exposure due to multiple acquisition phases.

CT has also been shown to offer information on LV function and tissue characterisation. Given the advantages of CT in terms of fast acquisition times and broad availability, further developments and expanded clinical applications might be expected in the future [80]. 

### Nuclear Imaging

In routine clinical practice, nuclear imaging is used sparingly in TTS because of limited availability, cost, and exposure to ionising radiation; its use is therefore generally reserved for selected patients. Nevertheless, it provides valuable mechanistic insights particularly into sympathetic dysregulation and the contribution of inflammation.

Among SPECT techniques, cardiac sympathetic innervation can be assessed with iodine-123 metaiodobenzylguanidine (123I-MIBG) imaging [96]. In TTS, hypokinetic myocardial segments typically show reduced 123I-MIBG uptake, indicating impaired sympathetic nerve function [97]. These abnormalities may persist for years after the acute episode and have been linked to a higher risk of arrhythmias, likely reflecting regions of denervation [98]. PET can be considered as a complementary modality (e.g., metabolic/inflammatory assessment), but its application in TTS remains mainly academic at present [99]. 

## Prognosis and Long-term Implications

Patients with TTS typically experience complete recovery of ventricular function within weeks to months following the acute phase, and are generally considered to have a favourable long-term prognosis [8, 13]. However, recent studies have shown that long-term mortality in TTS patients is higher than that observed in the healthy general population [3, 16, 100]. Mortality in TTS patients is strongly influenced not only by the degree of myocardial dysfunction but also by the presence of comorbidities [13, 101]. Adverse outcomes have been linked to cardiac arrest, cardiogenic shock, or acute HF at presentation, as well as elevated B-type natriuretic peptide (BNP) [2, 3, 102–104]. In addition, left-ventricular free-wall rupture, although rare (~ 0.5–1%), should be recognised as a life-threatening complication with high mortality [27]. Prognosis is particularly poor in elderly patients, men and non-white individuals [101, 103]. Additionally, atypical TTS forms with myocardial involvement extending beyond the apical region have been identified as independent predictors of complications and mortality during follow-up [103].

While most patients recover completely, a subset continues to experience persistent symptoms, such as dyspnoea, palpitations, fatigue and chest pain, even in the absence of ECG abnormalities or myocardial dysfunction [105]. This clinical picture suggests ongoing myocardial inflammation, which may persist for months after the acute event, as indicated by persistent abnormalities of myocardial tissue on CMR [33, 89, 90, 106]. The severity of inflammatory response during the index episode has been correlated with an increased risk of adverse outcomes during follow-up, as evidenced by elevated serum levels of IL-6 in patients with long-term poor prognosis [76].

Chronic myocardial inflammation may also underlie the risk of TTS recurrence, which has been reported in 0% to 14% of patients and is associated with worse prognosis, including a higher likelihood of fatal arrhythmias and death [10, 16, 107]. Recurrent episodes typically mirror the clinical presentation of the initial event, implying a reactivation of the same pathological mechanism [103]. The risk of recurrence is higher among younger and female patients, particularly those exposed to emotional triggers or with a history of psychiatric illness requiring psychotropic medications [13]. Furthermore, patients presenting with markedly elevated troponin levels during the initial episode appear to be at greater risk of recurrence, similar to those with lower LVEF [104]. The latter group has shown to have an increased inflammatory response, suggesting a potential correlation between the severity of inflammation and the extent of myocardial injury [104, 108]. This is supported by significantly elevated levels of C-reactive protein (CRP) – established biomarker of inflammation- observed in patients with TTS and reduced LVEF [108]. CRP has also been independently associated with an increased risk of TTS recurrence [108]. Elucidating the role of inflammation in the pathogenesis of TTS and its association with the risk of adverse events may be crucial for developing targeted therapeutic strategies aimed at improving prognosis, particularly in high-risk patient subsets.

## Current Treatment

Due to the absence of RCTs and the heterogeneous presentation of TTS, current treatment strategies are extrapolated mainly from HF guidelines and tailored to clinical presentation [109, 110]. Management is broadly divided into acute and chronic phases, with an emphasis on supportive care, hemodynamic stabilization, and symptom management.

### Acute Management

The acute phase focuses on haemodynamic stabilization and treatment of complications such as cardiogenic shock, arrhythmias, and thromboembolism.

The initial management of TTS involves close haemodynamic monitoring, initiation of medications similar to those used in ACS until this diagnosis is excluded, and adherence to standard advanced life support (ALS) protocols [111]. 

#### Acute Heart Failure Management

Acute HF in TTS is managed according to standard HF protocols, following current guidelines such as those of the ESC [110] and the American College of Cardiology [112]. Initial therapy focuses on oxygen and respiratory support as required, alongside strategies to reduce preload and afterload using diuretics and vasodilators where appropriate. When patients are hemodynamically stable, β-blockers may be considered to attenuate sympathetic overactivity and reduce heart rate, while ACEi or ARBs are used for afterload reduction. Diuretics are indicated when pulmonary congestion is suspected or when persistent oxygen requirements are likely attributable to volume overload. Receiving specialist HF care during hospitalization improves guideline-based therapy use and reduces diuretic-only discharge and mortality, especially in patients with reduced LVEF [113, 114].

#### Cardiogenic Shock

Although rare, cardiogenic shock requires prompt intervention. There are some reports that the incidence of cardiogenic shock can be as high as 5–10% [13, 115]. The decision on appropriate therapy largely depends on the presence of LVOT obstruction. Additionally, inotropic agents should be used with caution due to the potential for exacerbation of catecholamine-mediated toxicity and the potential risk of LVOT obstruction [116]. In patients with refractory hypotension and signs of end-organ damage despite inotropes, vasopressor and mechanical circulatory support (MCS) should be considered, including intra-aortic balloon pump, Impella, or extracorporeal membrane oxygenation (ECMO) [1, 117]. The calcium-sensitizing inodilator levosimendan has been shown to improve the acute course of TTS and may be particularly useful in the presence of LVOT obstruction or when other inotropic options are contraindicated [118]. In general, the management of TTS with LVOT obstruction parallels that of hypertrophic cardiomyopathy (HCM) with LVOT obstruction. In the absence of pulmonary oedema, fluid resuscitation may be beneficial, along with β-blockers therapy to reduce the obstruction [119]. 

#### Arrhythmias

The presence of arrhythmias in conjunction with TTS has been noted over the years. It includes asystole, pulseless electrical activity, complete sinoatrial and atrioventricular block, ventricular tachycardia (VT), and ventricular fibrillation (VF) [119, 120]. Ventricular arrhythmias, in the form of VT and non-sustained VT, are by far the commonest and most likely to occur in the acute phase. QT prolongation is common and may predispose to Torsades de Pointes (TdP), necessitating careful electrolyte management and QTc monitoring. Ventricular pacing can also be used to abort prolonged QT interval-related ventricular arrhythmia. Management of QT prolongation is empirical and based on well-established guidelines such as the European Society of Cardiology (ESC) guidelines on ventricular arrhythmias (VA) and sudden cardiac death (SCD). Otherwise, current literature does not provide TTS-specific management or pharmacotherapy in this particular indication.

As most patients will tend to receive β-blockers therapy as part of HF management, this is also effective management for the prevention of primary and secondary VA. Routine use of other antiarrhythmic agents is generally not recommended. Avoidance of QT-prolonging drugs is also encouraged due to the increased prevalence of QT prolongation, as is the careful monitoring and replacement of electrolytes such as magnesium and potassium to reduce any proarrhythmic substrate.

Although rare, in the presence of high-degree atrioventricular (AV) block (AVB), inotropes and permanent devices should be avoided [119]. 

### Chronic Management

Recovery of systolic function usually occurs within days to weeks; however, long-term management should not be neglected due to risks of recurrence, persistent symptoms, and HF.

#### Heart Failure Therapy

There is no clear consensus on the role of long-term neurohormonal blockade in TTS. β-blockers, ACEi, or ARBs are often prescribed empirically, particularly if LV dysfunction persists. However, their role in preventing recurrence remains unproven. While some meta-analyses indicate that ACEi/ARBs treatment may reduce recurrence and mortality rates, the absence of RCTs means that the long-term benefits remain uncertain [121, 122]. Conversely, although β-blockers represent one of the four pillars of modern HF therapy, their role in TTS remains uncertain. Recently, data from the GErman Italian Spanish Takotsubo Registry (GEIST) suggest that β-blocker therapy is associated with lower follow-up mortality, but not with a reduced risk of TTS recurrence [123]. In an extensive observational study of 2,672 patients, Isogai et al. demonstrated that β-blockade in TTS did not lower 30-day inpatient mortality [124]. This was also reiterated in the Spanish TTS registry, RETAKO [125] and from the SWEDEHEART registry [126]. Variation in LV function recovery also makes the duration of long-term therapy outside of the index hospital presentation challenging. In clinical practice, ACEi or ARBs therapy is typically continued for at least four weeks following an episode of TTS [127] to allow for adequate restoration of systolic function. The treatment could be extended for up to two to three months, with follow-up echocardiography guiding the duration of therapy. For individuals with coexisting conditions such as hypertension, CAD, or chronic HF, longer-term and potentially indefinite, use of ACEi/ARBs therapy is often considered appropriate beyond recovery from TTS [128].

In this context, sacubitril/valsartan (ARNi) has also been explored as a potential option. In an observational analysis, ARNi use was associated with lower inflammatory markers (e.g., WBC, IL-6) at 30 days and better survival at 30 days and 1 year versus no ARNi [129]. Complementary preclinical data in isoprenaline-induced TTS-like models show that sacubitril/valsartan attenuates inflammation via TLR4/NF-κB and reduces myocardial fibrosis via TGF-β/Smad, effects partly linked to augmented natriuretic-peptide signalling [129]. Taken together, these signals are hypothesis-generating; dedicated RCTs are needed to define efficacy, timing, and duration of ARNi therapy in TTS.

#### Anti-inflammatory Therapies in TTS

Inflammation plays a central role in the pathophysiology of TTS, providing a rationale for anti-inflammatory therapies. Agents targeting the IL-1 pathway, such as anakinra (a recombinant IL-1 receptor antagonist) and canakinumab (a monoclonal antibody against IL-1β), may attenuate inflammasome-driven injury by preventing IL-1β release and downstream activation of IL-6 and TNF-α [130]. These drugs have demonstrated cardio-protective effects in other inflammatory cardiac conditions, including myocarditis and HF, and could theoretically modulate the acute inflammatory burst observed in TTS [131]. 

Colchicine, which inhibits microtubule polymerisation and suppresses activation of the NLRP3 inflammasome, represents another promising candidate for mitigating sterile inflammation [132]. 

Cytokine-directed therapies, including anti-TNF-α agents and IL-6 receptor blockade with tocilizumab, may also counteract maladaptive immune activation and endothelial dysfunction, although no RCTs have directly tested them in TTS [133]. Tocilizumab, in particular, has shown short-term improvement in myocardial injury markers in acute coronary syndromes and could conceivably benefit TTS patients with persistently elevated IL-6 levels [134]. 

In addition, antioxidant and pleiotropic anti-inflammatory drugs such as SGLT2 inhibitors may reduce oxidative stress, improve endothelial function, and modulate macrophage polarisation toward a reparative phenotype [135, 136]. Preclinical data suggest that these agents attenuate catecholamine- and ROS-mediated cardiac injury, aligning with the proposed mechanisms of TTS.

An emerging line of investigation focuses on calcineurin inhibition as a strategy to modulate inflammation and oxidative stress. In the CIT-DZHK29 trial (NCT05946772), funded by the German Centre for Cardiovascular Research, evaluating whether short-term administration of cyclosporine A (CsA) can reduce myocardial damage and inflammation in patients with TTS. This randomized, double-blind, multicentre study includes patients with acute TTS at high risk of complications, who receive high-dose cyclosporine A or placebo in addition to standard HF therapy. The trial aims to determine whether transient immunosuppression can improve myocardial recovery and long-term outcomes by dampening the excessive stress-related inflammatory response. If successful, it will represent the first controlled clinical evidence supporting targeted anti-inflammatory therapy in TTS.

To date, however, evidence remains limited to case reports and small observational studies, with no published RCTs supporting the routine use of immunomodulatory therapy in TTS. Future research should clarify whether immunomodulatory treatment can move beyond symptomatic relief to become a disease-modifying strategy in TTS.

#### Anticoagulation

Clinical consensus drives the guidance on anticoagulation therapy. Prevalence of ventricular thrombus is reported to be around 1.3%−2.5% in extensive registry data [13, 137]. Although not particularly high, it is clinically significant due to potentially severe, debilitating consequences and increased mortality. Despite this, there is no clear consensus on early initiation of anticoagulation in the presence of severe LV impairment without evidence of thrombus on echocardiography or CMR. There have been reports that early initiation in the first 10 days reduced the incidence of thrombus [127]. However, anticoagulation therapy is usually initiated in most centres on confirmation of LV thrombus to prevent systemic embolization. Anticoagulation should be continued for 3 months in patients with documented apical thrombus or significantly reduced LVEF, with decisions on continuing therapy guided by follow-up imaging [137, 138]. 

#### Psychiatric Interventions

Up to 50% of patients report a preceding emotional trigger, and anxiety or depression is common [119]. Psychological support, cognitive-behavioural therapy, and pharmacological interventions (e.g., SSRIs) may reduce recurrence risk and improve quality of life [139]. Studies focusing on psychosocial aspects of TTS recovery, such as physical exercise and mental well-being, are also underway, see Table 1.

## Future Directions

Advances in artificial intelligence, particularly in multi-modal data integration, could enable early identification of high-risk patients, prediction of recurrence, and personalised therapeutic strategies by combining imaging, biomarker, and clinical profiles into robust predictive models [140]. 

On the therapeutic side, several paths emerge from the inflammation-centred biology of TTS. Mechanistically, stress-triggered catecholamine excess, oxidative stress (e.g., NOX4), and NLRP3–calcineurin/NFAT signalling suggest testable targets such as antioxidants, NADPH-oxidase and inflammasome inhibitors, and calcineurin/NFAT modulators; the acute cytokine response and macrophage infiltration point to anti-cytokine strategies (e.g., IL-1 or IL-6 blockade) and approaches that favour reparative macrophages polarisation.

In parallel, drugs traditionally used in microvascular angina, such as calcium-channel blockers, ranolazine, ivabradine, nicorandil, and vasodilatory β-blockers, warrant evaluation for their potential to improve coronary microvascular perfusion, reduce endothelial dysfunction, and limit catecholamine-mediated oxygen demand mismatch. By enhancing microvascular flow and attenuating vasospasm, these agents may indirectly mitigate the ischemia–inflammation loop that sustains myocardial stunning in TTS. Metabolic modulators, particularly SGLT2 inhibitors, may further provide anti-inflammatory and antioxidant effects, improving mitochondrial efficiency and cellular resilience to oxidative stress. Finally, neurohumoral modulators such as clonidine or other central sympatholytic agents could theoretically restore autonomic balance by dampening the excessive sympathetic discharge that triggers the syndrome [141]. 

Current ongoing RCTs evaluating different aspects of TTS management, including pharmacotherapy, are listed in Table 2. Ongoing clinical RCTs are exploring diverse therapeutic strategies in TTS. These include the pragmatic β-TAKO trial (NCT06509074; EU-CT 2023–510213-25-01) testing β-blockers in acute TTS; SAFT (NCT05768542) evaluating metoprolol’s effects on sympathetic and vascular function; and the Optimized Pharmacological Treatment for Broken Heart (Takotsubo) Syndrome study (NCT04666454). The CIT-DZHK29 trial (NCT05946772) targets the calcineurin pathway using cyclosporine A, while NACRAM (ACTRN12616000781448) investigates N-acetylcysteine followed by/or ramipril. The ongoing EVEREST trial (ISRCTN18302602) is the first large-scale RCT designed to test whether RASi can reduce mortality, major adverse cardiovascular events, and recurrence after TTS. Additional studies include BROKEN-SWEDEHEART, evaluating adenosine, dipyridamole, and apixaban for microvascular and thrombotic mechanisms; haemodynamic support with levosimendan in low-EF TTS (Polish registry); and a β-blocker class comparison (Spanish registry). Non-pharmacologic programmes, such as structured cardiac rehabilitation with psychological support (NCT04425785) and an upcoming NYU deep-breathing intervention, aim to address autonomic and psychosocial determinants of the syndrome. Current evidence still relies largely on small observational cohorts, and definitive large-scale RCTs are lacking. Nevertheless, biomarker-guided, inflammation-targeted, and autonomic-modulating strategies, ideally coupled with AI-enabled phenotyping, may pave the way for truly personalised therapy in TTS [45].

**Table 2:** Current trials of Pharmacological and non-pharmacological management of Takotsubo cardiomyopathy

Trial Name	ID	Design	Primary Endpoint / Start	Why it Matters
Pharmacologic/Device-style Interventions				
β-Tako Trial – β-blockers in Takotsubo Syndrome	NCT06509074; EU-CT: 2023–510213-25-01	Pragmatic, multicentre, randomised β-blockers vs. no β-blockers; open-label with blinded endpoint assessment.	Wall motion score index at 7 days.	First adequately powered RCT to test whether β-blockers help in acute TTS.
The N-AcetylCysteine and RAMipril in Takotsubo Syndrome Trial (NACRAM)	ACTRN12616000781448	Multi-centre, randomised, placebo-controlled trial, sequentially testing early use of intravenous N-acetylcysteine (NAC), followed by/or oral ramipril for 12 weeks.	Resolution of myocardial oedema on cardiac magnetic resonance imaging (CMR), improvements in LV systolic function as measured by global longitudinal strain (GLS) on echocardiography, quality of life, and inflammatory markers Start: 2016	The first prospective study to help definitively evaluate a therapeutic option in acute attacks of TTS
SAFT – Sympathetic and Vascular Function in Takotsubo Syndrome	NCT05768542	Mechanistic trial assessing metoprolol’s effect on sympathetic nerve activity and vascular function in TTS.	–	Targets the catecholamine/sympathetic hypothesis directly.
Optimized Pharmacological Treatment for Broken Heart (Takotsubo) Syndrome	NCT04666454	Interventional study aiming to define an optimised multi-drug regimen for TTS.	–	Attempts to systematize HF-style therapy specifically for TTS.
Cyclosporine in Takotsubo syndrome (CIT) trial (CIT-DZHK29 Trial)	NCT05946772	Pilot multicenter double-blinded randomized placebo-controlled trial (RCT) to investigate the impact of CsA bolus therapy in patients suffering from acute TTS	The reduction myocardial damage quantified by AUC of a centrally measured high-sensitive cardiac Troponin T (hs-cTnT) over 72 h	The results of this trial may reveal CsA as a first pathophysiology-driven treatment option of TTS
Study of Levosimendan (± Glucose Monohydrate) in Low-EF Takotsubo Syndrome	EU registry (Poland)	Levosimendan vs. comparator in acute low-EF TTS.	Start: 22 Nov 2024	Tests an inodilator in the acute, haemodynamically compromised phenotype.
β-blocker Class Comparison Trial in TTS	EU registry (Spain)	Head-to-head class comparison of β-blockers (selective vs. non-selective vs. α/β-blockers).	Start: 8 Jul 2024	May clarify whether specific β-blocker pharmacodynamics (e.g., α-blockade, NO release) are advantageous or harmful.
Adenosine / Dipyridamole / Apixaban in TTS - BROKEN-SWEDEHEART	Nordic, multicentre (EU)	Multiple therapeutic arms, including adenosine and antithrombotic strategies.	Start: 15 Dec 2020	Explores coronary microvascular dysfunction and thrombotic risk modulation.
Cyclosporine in Takotsubo syndrome (CIT-DZHK29)	NCT05946772	Pilot multicenter double-blinded randomized placebo-controlled trial (RCT)	Reduction myocardial damage quantified by AUC of a centrally measured high-sensitive cardiac Troponin T (hs-cTnT) over 72 h.	This RCT may reveal CsA as a first pathophysiology-driven treatment option of TTS and enable a phase III follow-up trial powered for clinical outcome parameters as primary endpoint
A Randomised Controlled Trial of Renin-Angiotensin System Inhibition for Reduction of Cardiovascular Events after Takotsubo Cardiomyopathy (EVEREST Trial)	ISRCTN18302602	Two-arm prospective randomised open label blinded endpoint multicentre trial	all-cause death or hospitalisation for either heart failure, myocardial infarction, stroke or recurrent takotsubo cardiomyopathy. Start: January 2025	aims to determine whether renin–angiotensin system inhibitors, widely used in post-infarction and heart failure care, can improve survival and prevent recurrence in TTS. Its results could finally provide the first evidence-based pharmacological strategy for this condition.
**Non-pharmacologic Rehabilitation / Behavioral Interventions**				
Physical Exercise and Mental Well-being Rehabilitation After Acute TTS	NCT04425785	Structured cardiac rehabilitation + psychological programme post-TTS.	–	Addresses the high psychosocial stress burden and potential autonomic dysregulation.
NYU “Deep Breathing” Study in TTS (upcoming)	Institutional announcement (not yet on ClinicalTrials.gov)	Autonomic/breathing intervention to blunt sympathetic surges.	–	Targets the stress–autonomic axis non-pharmacologically.

**Key**: AUC: area under the curve; CMR: cardiac magnetic resonance; CsA: cyclosporine A; EF: ejection fraction; GLS: global longitudinal strain; HF: heart failure; hs-cTnT: high-sensitivity cardiac troponin T; LV: left ventricle; NAC: N-acetylcysteine; NCT: National Clinical Trial (ClinicalTrials.gov identifier); NO: nitric oxide; RCT: randomised controlled trial; TTS: Takotsubo syndrome

## Conclusions

TTS remains a complex and heterogeneous entity with substantial gaps in understanding of its pathogenesis and long-term impact. While supportive care remains the cornerstone of treatment, increasing recognition of inflammatory and neurohumoral mechanisms opens new frontiers for targeted therapy. Future research should focus on subphenotype identification, risk stratification, and testing novel therapies through prospective RCTs. This approach may help pave the way toward moving beyond symptom management to achieving disease modification and recovery in TTS.

## Data Availability

No datasets were generated or analysed during the current study.
